# An anode catalyst support for polymer electrolyte membrane fuel cells: application of organically modified titanium and silicon dioxide

**DOI:** 10.1039/c9ra04862f

**Published:** 2019-08-07

**Authors:** Marek Malinowski, Agnieszka Iwan, Agnieszka Hreniak, Igor Tazbir

**Affiliations:** Hydrogen South Africa Systems and Validation Centre, SAIAMC, University of the Western Cape Robert Sobukwe Road, Bellville Cape Town South Africa malinowski.137@gmail.com; Military Institute of Engineer Technology Obornicka 136 Str. 50-961 Wroclaw Poland; Electrotechnical Institute, Division of Electrotechnology and Materials Science M. Sklodowskiej-Curie 55/61 50-369 Wroclaw Poland

## Abstract

This work describes an attempt to improve the physical and electrochemical parameters of PEM fuel cells that have electrodes modified by titanium and silicon dioxides. A customized design of membrane electrode assemblies was proposed which is characterized to have an around 6 times higher concentration of catalyst at the cathode side (2.0 mgPt cm^−2^) in order to investigate the influence of anode catalyst support treatment. Anode catalyst support materials were modified using pristine TiO_2_ and TiO_2_–SiO_2_–VTMS – the composite was crosslinked with the aid of vinyltrimethoxysilane. Surface area and porosity analysis was carried out with the aid of BET, BJH, t-plot and Horvath–Kawazoe (H–K) theories for particular components of the support materials and their catalyst mixtures. The experiment revealed a positive influence of TiO_2_–SiO_2_–VTMS (BET 321.9 m^2^ g^−1^, BJH 3.7 nm) on the anode catalyst layer in terms of surface area (3-times increase, 75 m^2^ g^−1^) and average pore size (decrease from 25.3 to 15.7 nm). Additionally, favourable microporosity (pores less than 2 nm) was introduced to the material according to the H–K analysis results (10.3 m^2^ g^−1^, 0.65 nm). Electrochemical experiments, which include polarization curves, electrochemical impedance spectroscopy and cyclic voltammetry, have demonstrated the change of behaviour for the fabricated fuel cells with modified anodes against the reference sample. The mitigation of charge and mass transfer resistance (by 15–20%, 50 mV at 200 mA cm^−2^), the improvement of power density (up to 72%, 217 mW cm^−2^) and a better exposure of the catalyst to the reactants of an electrochemical reaction were observed for fuel cells modified by both pristine TiO_2_ and the hybrid TiO_2_–SiO_2_–VTMS-based compound.

## Abstract 2

TiO_2_ and SiO_2_ act successfully as an anode support material despite their poor electrical properties providing the improvement of fuel cell electrochemical parameters.

## Graphical abstract

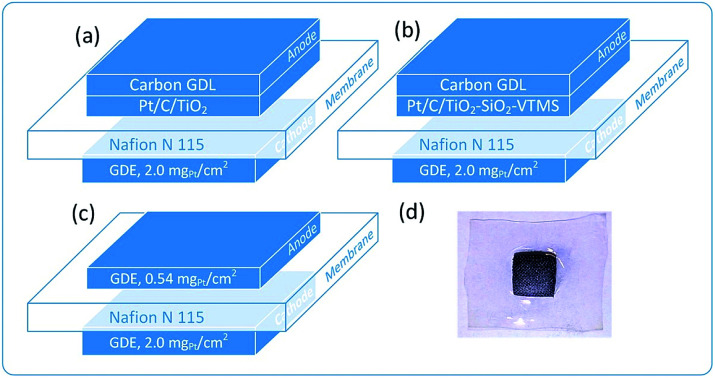

## 1. Introduction

The performance and durability of polymer electrolyte membrane fuel cells (PEMFCs) are mainly affected by two factors: the physicochemical properties of proton exchange membranes (PEM) and the rate of the ORR reaction (Oxygen Reduction Reaction) at the cathode side. These aspects attract many scientific studies including materials research and investigation of the various phenomena influencing fuel cell operation such as activation losses, ionic conductivity and decay mechanisms.^1^ Despite the sluggish kinetics of the cathode reaction and less significant problems with the ageing processes happening at the hydrogen side, the modification of the anode is also a matter of great concern to accelerate the commercialization of fuel cells in diverse applications and various conditions of operation.

Due to the fast rate of HOR reaction (Hydrogen Oxidation Reaction), around six orders of magnitude faster than ORR,^2^ negligibly-small voltage losses are present at the anode side for Pt catalyst loading down to 0.05 mg cm^−2^.^3,4^ However, at a very low loading, there is a high risk of severe contamination which might come from the chemical compounds carried by the fuel. The contaminants such as carbon oxide, hydrogen sulfide or ammonia can interact with platinum particles creating strong, practically irreversible chemical bonds, consequently decreasing the electrochemical surface area.^1^ Due to this reason, a very low anode catalyst loading may not be preferred. On the other hand, high catalyst loading at anode side accelerates the phenomenon called reverse-current decay mechanism.^5^ This mechanism produces cathode layer degradation *via* carbon corrosion of the catalyst support happening if oxygen is present at anode side in the fuel cell stack (in most cases during start-stop cyclings). The reduction of reverse current is one of the methods to suppress degradation processes. The adjustment of catalyst amount and modification of catalyst support is therefore appropriate approach towards preparation of durable and cost-effective anode materials. One of the approaches is an attempt to modify catalyst support applying titanium dioxide as a replacement of carbon-based support materials. For instance, in ref. 6 authors proposed a new strategy for alleviating the reverse current phenomenon using a method named atmospheric resistive switching mechanism (ARSM). In this method, the resistance of anode catalyst layer composed of Ta-doped TiO_2_-supported Pt varies depending on the gas atmosphere: in the air present at the anode side, the value of cell resistance is approximately one order of magnitude larger than in hydrogen (1.2 *vs.* 0.13 Ω cm^2^) which helps minimizing destructive corrosion of the cathode.

Many other scientific papers describe the application of titanium dioxide in fuel cells^7^ including its application to membranes and cathodes. TiO_2_ is investigated despite the fact, the pristine TiO_2_ is a semiconductor, *i.e.* it has the band gap of 4.85 eV and electrical conductivity as low as 10^−13^ Ω^−1^ cm^−1^ at 298 K.^8^ High conductivity of TiO_2_-supported catalyst layer can be created by introduction of crystal lattice defects to the TiO_2_,^9–11^*via* doping using various metals such as niobium, chromium or vanadium^6,12–14^ or preparing the mixture of TiO_2_ and various allotropic forms of carbon.^9,15–17^ For instance, in,^11^ fuel cell with titanium dioxide acting as a Pt support, achieved outstanding performance in comparison to Pt/carbon-based catalyst in terms of durability (electrochemical surface area of 11.0 *vs.* 3.8 m^2^ g^−1^ after 80 h of accelerated stress test). At the same time the sample obtained comparable initial power density 0.84 *vs.* 0.94 W cm^−2^. Similar results were described in ref. 15, however the authors applied also carbon nanotubes (CNT) in order to increase the electrical conductivity. Experimental results revealed that CNT-Pt/TiO_2_ nanofiber-based electrode shows enhanced performance with the maximum power density of 567 mW cm^−2^ compared to commercial Pt/carbon (461 mW cm^−2^) with a significant durability at harsh conditions of 120 °C and RH 40%. The effect named as Strong Metal Support Interaction (SMSI) was demonstrated as an additional advantage of using TiO_2_, based on which the platinum dissolution and agglomeration of its particles are significantly limited leading to a great durability of fuel cell electrodes. The SMSI effect was also presented in ref. 10 for Nb–TiO_2_ utilized as Pt support. In this work a significant increase in the activity of Pt/TiO_2_ for oxygen reduction reaction was shown along with the improvement of catalyst stability.

Fuel cell electrode materials are also modified using silicon. Carbides of this element were considered mainly due to good electrochemical and physical stability acting as a support for platinum particles.^18–20^ Various applications require a special design of fuel cell stack that allows on a self-humidification process. It is relevant to applications where space and weight must by maximally limited (humidifiers are not needed) or for open-cathode fuel cells which are susceptible to sudden changes of current density and temperature. Silicon dioxide, due to its hydrophilic properties, seems to be a good candidate for the electrode modification despite its poor electrical conductivity. For instance, in ref. 21 J. I. Eastcott and E. B. Easton examined the physical and electrochemical characteristics of sulfonated silica ceramic carbon electrodes for PEMFC working at low humidity. The silica-based cathodes were able to maintain stable fuel cell performance down to 20% RH, and had better performance than the Nafion-based cathode. In this work, authors concluded that the presence of sulfonated silane in the cathode catalyst layer may aid in water retention and promote back-diffusion of water from the cathode to the membrane to improve MEA hydration. C.-L. Lin *et al.*^22^ investigated silicon dioxide used as an anode catalyst layer to improve the performance of a self-humidifying proton exchange membrane fuel cells. Using just 0.01 mg cm^−2^ of sulfonated SiO_2_, the electrical performance of fuel cells was increased 29% and 59% when the fuel cell reaction gases were humidified at 70 °C and 50 °C, respectively. Similar conclusions were found in ref. 23, where various concentrations of SiO_2_ were investigated. Silica nanopowders added to catalyst layers contributed to self-humidification effect improving the performance of fuel cell samples.

This work aims at the investigation of fuel cells containing both compounds *i.e.* TiO_2_ and SiO_2_, and additionally vinyltrimethoxysilane (VTMS) acting as a binder. VTMS was used in organic modification process of titanium and silicon dioxide. Synthesized modifiers were applied to anode catalyst supports. Fuel cells (FC) containing various concentrations of TiO_2_ or TiO_2_–SiO_2_–VTMS in catalyst layer (CL) were prepared in order to perform electrochemical characterisation. Additionally, fuel cell with reference catalyst layer used as an anode was also fabricated. Unique properties of TiO_2_ and SiO_2_ change the characteristics of investigated samples in terms of performance, durability and also production costs due to lower surface density of platinum catalyst which is required for efficient operation.

The goal of this work was to show that it is possible to create efficient and not expensive PEMFCs with reduced amount of Pt by incorporation of cost-effective hybrid organic-inorganic or inorganic compounds obtained by sol–gel method.

## 2. Experimental section

### 2.1. Synthesis of TiO_2_

Anatase crystals of titanium dioxide were synthesized based on sol–gel method using titanium(iv) isopropoxide (TIPO) as a titanium precursor. The synthesis procedure is as follows. TIPO (4.5 ml) was dissolved in ethanol (21 ml) mixed with 3.5 ml of distilled water to obtain a titania solution. The solution was mixed using a magnetic stirrer in a plastic flask at room temperature for 6 h. During the process, the TiO_2_ powder was formed and once filtered it was dried at room temperature. Thereafter TiO_2_ powder was heated at 500 °C for one hour.

### 2.2. Synthesis of organically modified TiO_2_–SiO_2_

Vinyltrimethoxysilane (VTMS) was applied for organic modification of TiO_2_ and SiO_2_. VTMS is an organosilicon compound with the formula H_2_C=CHSi(OCH_3_)_3_. It was used in a form of colorless liquid. The powder of TiO_2_–SiO_2_–VTMS was obtained carrying out hydrolysis of titanium and silane precursors *i.e.* titanium(iv) isopropoxide (TIPO) and tetraethoxysilane (TEOS), respectively. According to the synthesis procedure, TIPO (2 ml), TEOS (2 ml) and VTMS (2 ml) were separately mixed with 96 vol% ethyl alcohol (7 ml) and distilled water (3.5 ml) at room temperature, for two hours using magnetic stirrer. NH_3_ (4 ml) was used as a catalyst of the synthesis reaction. Afterwards, particular solutions were mixed one with each other in order to obtain TIPO : TEOS : VTMS molar ratio of 1 : 1.3 : 2. The combined solution was stirred additionally for 5 hours at room temperature. Finally, the yielded material was precipitated in the form of white powder and dried at room temperature.

### 2.3. Preparation of catalyst layers and MEAs

Nafion N115 membranes (FuelCellsEtc) were used as a fuel cell electrolyte. Prior to the usage, 3 × 4 cm Nafion sheets were purified by boiling in deionized water for one hour and then rinsing in 3.75% hydrogen peroxide for an hour. Thereafter, the membranes were activated in 0.2 M H_2_SO_4_ solution at boiling temperature also for one hour. Finally, they were rinsed several times in deionized water to remove any residual contaminants. To make Membrane Electrode Assemblies (MEAs), commercial gas diffusion electrodes (MLGDE 2.0 mg.cm^−2^, FuelCellsEtc) were selected as a cathode and Gas Diffusion Electrodes (GDEs) were prepared as an anode. Both electrodes were cut to maintain the equal area of 1 cm^2^. Catalyst mixtures intended for anodes were fabricated by mixing of particular catalyst components in the way to obtain the uniform distribution of the platinum particles and two types of modifiers (TiO_2_ and TiO_2_–SiO_2_–VTMS). This was done according to the following procedure. Highly conductive Vulcan XC72R carbon black (0.33 g) was used as anode supporting material to prepare mixtures in individual 25 cm^3^ beakers. Pt black powder (0.140 g) was first mixed with a small amount of deionized water (to avoid catalyst burning) and then carbon black was added to obtain a 30% Pt/C mass fraction. Next, ethanol (96% vol.) was used in the amount of *ca.* 15% volume ratio. The mixtures were prepared by applying the ultrasonication stirrer for about 25 minutes. At this stage two modifiers *i.e.* TiO_2_ and TiO_2_–SiO_2_–VTMS were added, both in the form of powder. One catalyst mixture was also prepared as a reference unit. To investigate the contribution of TiO_2_ and TiO_2_–SiO_2_–VTMS to the anode behavior, three different amounts of these modifiers were investigated, *i.e.* 0.05 g, 0.1 g and 0.2 g. The given amounts were found considering the maximum concentration of a non-conductive additive in electrode structure to keep its good electron conductivity. Next, catalyst mixtures were again stirred and mixed for about 10 minutes. Finally, 20 wt% Nafion solution (Sigma-Aldrich) was added into each beaker in the amount of 1.16 g followed by the refrigeration of mixtures to the temperature of about 0 °C.

As a result, seven catalyst inks were obtained, (a) one set of TiO_2_-modified catalyst ink (CL_T50, CL_T100, CL_T200), (b) one set of TiO_2_–SiO_2_–VTMS-modified catalyst ink (CL_TS50, CL_TS100, CL_TS200), and (c) the reference catalyst ink (CL_Ref).

GDL papers with hydrophobic treatment (H2315 C4 Freudenberg Fuel Cell Products) were cut out (4 cm^2^), weighed and used to made gas diffusion electrodes (1 cm^2^) by casting of the prepared catalyst inks (tape casting). Obtained GDEs were dried at a temperature of 25 °C for one hour and then for 15 minutes at 135 °C and finally weighed again. The mass difference between GDL and GDE was used to calculate the amount of Pt, TiO_2_ and TiO_2_–SiO_2_–VTMS incorporated in particular GDEs. Details with regard to constructed electrodes are given in Table 1.

**Table 1:** Concentrations and surface densities of platinum catalyst and particular modifiers used in the process of anode catalyst layers preparations

Catalyst layer^a^	Content of CL	Pt amount [wt%]/[mg cm^−2^]	Modifier amount [wt%]/[mg cm^−2^]
CL_Ref	X	20.3/0.54	0/0
CL_TS100	TiO_2_–SiO_2_–VTMS	17.4/0.395	12.5/0.28
CL_TS50	TiO_2_–SiO_2_–VTMS	18.9/0.35	7.2/0.13
CL_TS200	TiO_2_–SiO_2_–VTMS	15.4/0.39	22.6/0.57
CL_T100	TiO_2_	17.4/0.38	12.4/0.27
CL_T50	TiO_2_	18.7/0.36	6.6/0.13
CL_T200	TiO_2_	15.8/0.34	22.1/0.48

a Where the numbers 50 and 200 mean double decrease and increase of the modifier's amount in comparison to 100.

**Fig. 1:**
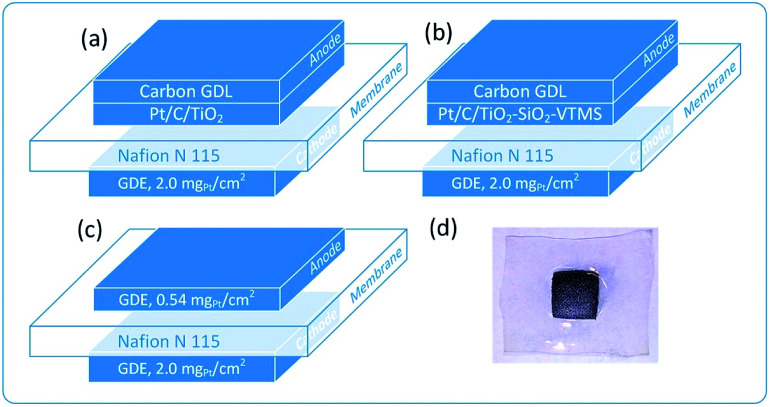
Architecture of polymer fuel cells investigated in this work: (a) TiO_2_-based anode, (b) TiO_2_–SiO_2_–VTMS-based anode, (c) reference fuel cell and (d) the picture of MEA after the experiment.

Each fuel cell (MEA) was constructed by pressing of activated membranes and electrodes at a temperature of 110 °C for 5 minutes under 30 bar of pressure. Respectively, six modified fuel cells were obtained: FC_T50, FC_T100, FC_T200, FC_TS50, FC_TS100, FC_TS200 and one reference FC_ref. Fig. 1 presents the architectures of the constructed PEMFC as well as a photograph of a typical MEA.

### 2.4. Material characterization

Surface area and porosity analyzer Micromeritics *ASAP2020* was used to calculate the physical parameters of prepared catalyst mixtures. The analysis was also performed for pristine components (TiO_2_, TiO_2_–SiO_2_–VTMS and Vulcan carbon black) to evaluate their impact on the anode behavior. Adsorption/desorption isotherms were determined at temperature of liquid nitrogen, from high vacuum pressure (10^−6^ Torr) to the relative pressure of 0.99 *p*/*p*^0^, where *p*^0^ is the saturation pressure of the adsorbate. Prior to the measurements, catalyst mixtures were degassed at 150 °C for 8 hours (maximum limit due to Nafion used), whereas particular components at elevated temperature of 350 °C for 8 hours to perform deep purification process (except organically modified TiO_2_–SiO_2_–VTMS, 150 °C). To calculate surface area for particular samples, Brunauer–Emmett–Teller (BET) theory was applied while in case of average pore diameter and volume as well as pore size distribution, Barrett–Joyner–Halenda (BJH) method was used. Based on obtained results, additional analysis of micropore structure was carried out for selected materials using Horvath–Kawazoe and t-plot methods. Gas diffusion electrodes were analyzed by scanning electron microscope (SEM) (Tescan *VEGA*/*SBH*) in order to investigate the effect of applied additives on surface morphology.

Polymer fuel cells (MEAs) were electrochemically investigated using 1 cm^2^ testing fuel cell fixture (Pragma Industries) with graphite single-serpentine monopolar plates. The research was carried out *in situ*, delivering to the measurement cell reactant or inert gases, depending on the method used. The samples were measured by cyclic voltammetry (CV), polarization curve (*J*–*V*) and electrochemical impedance spectroscopy (EIS). The equipment used in these experiments was: SI 1287 Electrochemical interface potentiostat/galvanostat (Solartron Analytical), ZS electronic load (H&H), Impedance/Gain-Phase analyzer SI 1260 (Solartron Analytical) and dedicated control programs.

## 3. Results and discussion

### 3.1. Physical characterisation of materials

Porosity and the surface area of fuel cell electrodes have significant influence on the rate of both electrochemical reactions. High porosity and large surface area accelerates hydrogen oxidation and oxygen reduction reactions in terms of improved reactants delivery, products removal, heat energy and electrical charges, and transfers of ions. The improvement of reaction rate may happen due to the increase of active catalytic sites *via* larger exposure of catalyst to the reactants in a three-phase boundary region. The pores of average diameter less than 2 nm (micropores) promote higher rate of electrochemical reaction, while pores larger than 50 (macropores) nm facilitate charges, reactants and products transfer. Therefore, it is intentional to maintain both structures of pores in the catalyst area and to limit the size of mesopores (2–50 nm) which are mainly responsible for negative effect of additional mass transfer resistance and reduction of electrode volumetric current.^24^ Fuel cell performance strongly depends on the kinetics of electrochemical reactions, thus the modification of catalyst support is one of the methods to improve it.

**Fig. 2:**
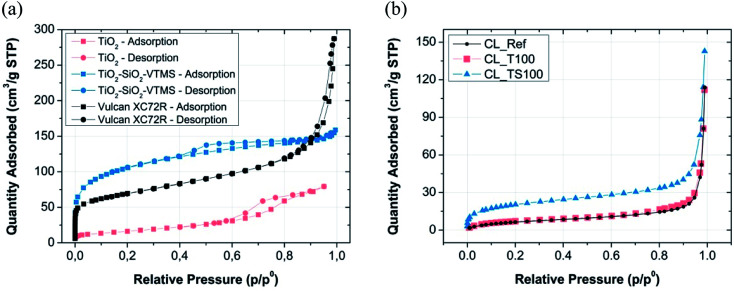
Nitrogen sorption isotherms for (a) particular CL components and (b) fabricated catalyst layer mixtures.

**Fig. 3:**
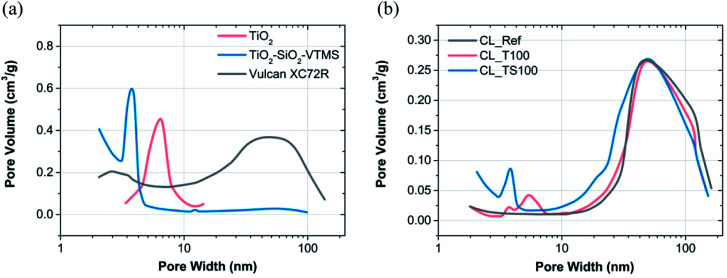
BJH pore size distribution for (a) material used to prepare catalysts mixtures and (b) reference and modified catalyst layers.

According to the graphs depicted in Fig. 2a and 3a particular electrode components that were used for catalyst mixture preparation are characterized by high surface area as well as high porosity.

Fig. 2a shows the shape of nitrogen adsorption/desorption isotherms for three various electrode components *i.e.* Vulcan carbon black, titanium dioxide and TiO_2_–SiO_2_–VTMS. Some characteristic properties can be retrieved by analyzing their low-pressure adsorption behaviour (*p*/*p*^0^ less than 0.01 where microporosity is revealed). Carbon black Vulcan XC72R isotherm is assigned to type I and II according to IUPAC classification.^25^ It suggests that this material has a fragmentary microporosity which is highly beneficial for fuel cell operation. TiO_2_–SiO_2_–VTMS behaviour is relevant at initial adsorption stage (also type I of isotherm) however, its isotherm reveals even better properties because the increase of nitrogen adsorption at low pressure is prolonged in comparison to Vulcan giving higher surface area and bigger volume of micropores (Fig. 3a). Moreover, it contains characteristic hysteresis which is concurrent with type IV of IUPAC classification. In this background, the third component TiO_2_ does not have unique structure of micropores, since the relative pressure immediately increases once adsorption begins which corresponds to a very low volume of micropores. Nevertheless, its isotherm is also characterized by a hysteresis.

Fig. 2b and 3b demonstrate result analysis of catalyst layers. TiO_2_ and reference catalyst layer isotherms are equivalent. The addition of TiO_2_ does not cause any improvement or deterioration of sorption phenomenon. Both isotherms are assigned to type II that describes materials composed of mainly macroporous structure. In the consequence of catalyst mixture preparation, micropores of Vulcan carbon black were closed and filled mostly by Nafion. Therefore at a very low pressure no significant adsorption could be observed. TiO_2_–SiO_2_–VTMS acts better than carbon black because some of its microporous structure is conserved once catalyst mixture is fabricated. This can be clearly seen from its isotherm – Fig. 2b.

Table 2 describes the results of BET and BJH calculations obtained for particular catalyst layers and their selected components. BET analysis was performed taking the isotherm data in the range from 0.05 to 0.2 *p*/*p*^0^ whereas BJH in the entire range of pressures excluding microporosity region (pressure less than 0.01 *p*/*p*^0^). Carbon black is characterized by surface area of 249.2 m^2^ g^−1^ and average pore width 10.1 nm according to the analysis. Preparation of catalyst mixture leads to a deterioration of physical parameters. Surface area of reference catalyst layer (CL_Ref), prepared on the basis of carbon black, is significantly reduced (25.9 m^2^ g^−1^) and average pore size is increased (25.3 nm). Simultaneously, cumulative volume of pores is decreased more than twice from 0.413 to 0.177 m^3^ g^−1^. TiO_2_–SiO_2_–VTMS with its superior physical properties (321.9 m^2^ g^−1^, 3.7 nm) applied with the concentration of just 12.5 wt% (Table 1) is capable to mitigate a negative effect of catalyst layer formation. Its catalyst mixture (CL_TS100) is characterized by 74.6 m^2^ g^−1^ and 15.7 nm of surface area and average pore width, respectively. Cumulative volume of pores for CL_TS100 is relatively low (0.146 m^3^ g^−1^), however the given value was calculated based on BJH theory that doesn't take into account micropores nor macropores of large size (the calculation of cumulative surface area and volume of pores was within the range of 1.7–300 nm). Upper result suggests that there is a large fraction of micropores implemented by TiO_2_–SiO_2_–VTMS to the catalyst layer. In contrast to CL_TS100, TiO_2_-modified catalyst layer (CL_T100) has low specific surface area (27.8 m^2^ g^−1^) and large average pore size (24.0 nm) – which are both comparable to the reference material. Such values result mainly from poor surface area of TiO_2_ (59.5 m^2^ g^−1^), which does not contribute to the improvement, however the influence on catalyst layer is slightly positive as its average pore width (6.2 nm) is relatively small giving additional space for adsorbate.

**Table 2:** BET specific surface area and characteristic parameters of pores (BJH theory, desorption isotherm) for given catalyst layers and selected components

Catalyst layer (CL)/component	BET	BJH – desorption		
	Surface area [m^2^ g^−1^]	Cumulative surface area of pores^a^, [m^2^ g^−1^]	Cumulative volume of pores^a^, [m^3^ g^−1^]	Average pore width^a^, [nm]
CL_Ref	25.9	28.0	0.177	25.3
CL_TS100	74.6	54.1	0.213	15.7
CL_T100	27.8	29.0	0.174	24.0
Vulcan XC72R	249.2	162.8	0.413	10.1
TiO_2_–SiO_2_–VTMS	**321.9**	**156.3**	**0.146**	**3.7**
TiO_2_	59.5	75.4	0.117	6.2

a In the range 1.7–300 nm.

Good results for CL_TS100 investigation were confirmed by the additional analysis with respect to the fraction of micropores. Horvath–Kawazoe (H–K) and t-plot methods were used to estimate the volume, size and surface area of micropores for TiO_2_–SiO_2_–VTMS, Vulcan carbon black as well as one catalyst mixture. Micropores are visible at a very low pressure of isotherms for only three materials (see Fig. 2). For H–K theory the calculation was done assuming a slit geometry of micropores and taking so called interaction parameter that is 3.73 r 10^−43^ erg cm^4^. For calculation of t-plot, in case of Vulcan carbon black, the thickness curve of Carbon Black STSA was used while for TiO_2_–SiO_2_–VTMS and its catalyst layer Harkins and Jura thickness curve was applied. Table 3 contains details of microporosity structure for given components.

**Table 3:** Physical properties of materials containing microporous structure

Component/catalyst layer	Theory		
	t-Plot	Horvath–Kawazoe	
	Surface area of micropores, [m^2^ g^−1^]	Average width of micropores, [nm]	Maximum micropore volume at *p*/*p*^0^ = 0.01, [cm^3^ g^−1^]
Vulcan XC72R	79.4	0.58	0.076
TiO_2_–SiO_2_–VTMS	**102.7**	**0.61**	**0.086**
CL_TS100	10.3	0.65	0.016

**Fig. 4:**
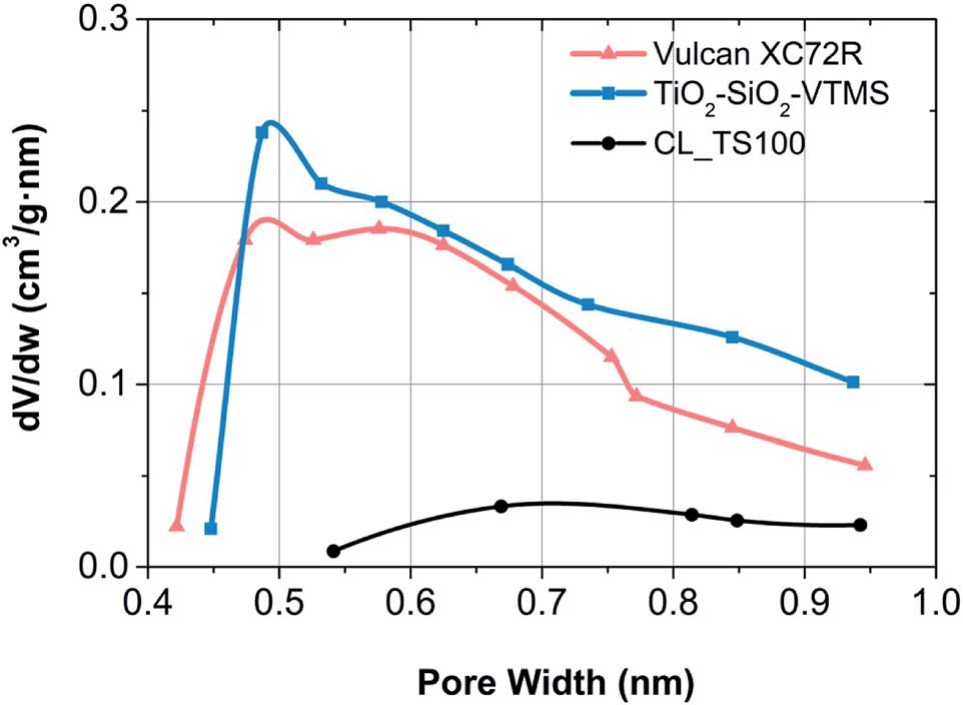
Micropore size distribution according to Horvath–Kawazoe theory for particular fuel cell electrode components.

Both components and the catalyst layer have comparable average pore width (around 6 nm), however according to the t-plot method, surface area of CL_TS100 is significantly lower than for carbon black as well as TiO_2_–SiO_2_–VTMS. Fabrication of catalyst layer leads to the decrease of specific surface area of pores which suggests that Nafion electrolyte was partially adsorbed within the range of micropores. Such a partial introduction of this polymer into the micropore structure is possible as, according to the cluster-network model, Nafion consists of an equal distribution of sulfonate ion clusters with a 40 Å diameter held within a continuous fluorocarbon lattice.^26^ This effect is also confirmed by Horvath–Kawazoe method giving lower micropore volume (0.016 cm^3^ g^−1^) for CL_TS100 in comparison to its pristine components (0.076 and 0.086 cm^3^ g^−1^, respectively). Micropore size distribution for Vulcan carbon black, TiO_2_–SiO_2_–VTMS and their catalyst layer is shown in Fig. 4. Derivative of pore volume (Akima Spline fitting) *versus* pore width reveals predicted micropore distribution at a given geometry of pores (slit), in the range from *ca.* 0.4 to 1.0 nm. Such configuration is advantageous for hydrogen oxidation reaction due to better exposure of catalyst site and improved proton transfer because of hydrophilic properties of SiO_2_.

**Fig. 5:**
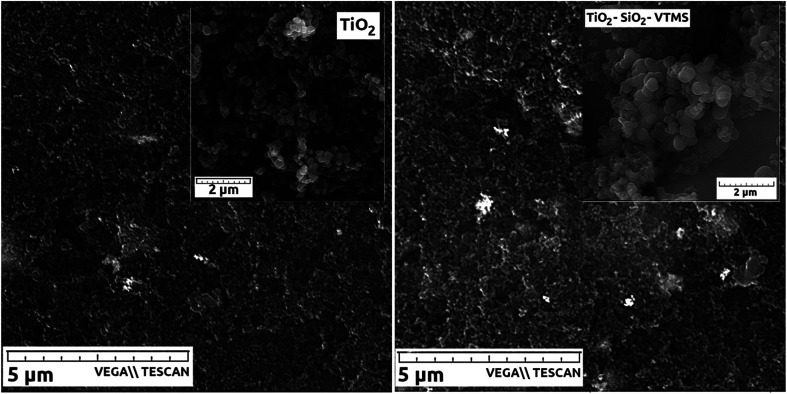
SEM micrographs of catalyst layers: CL_T100 (left), CL_TS100 (right). Insets show SEM pictures of compounds used to modify catalyst layers.

Fig. 5 depicts SEM micrographs of catalyst layers incorporating TiO_2_ and TiO_2_–SiO_2_–VTMS that show the uniform distribution of particular catalyst components. Two insets of SEM pictures for pristine TiO_2_ and TiO_2_–SiO_2_–VTMS particles^27,28^ reveal the structure of both modifiers. These compounds occur in the form of round-shape particles that undoubtedly facilitates the fabrication process of catalyst layers.

### 3.2. Electrochemical characterization

#### 3.2.1. The performance of fuel cells

**Fig. 6:**
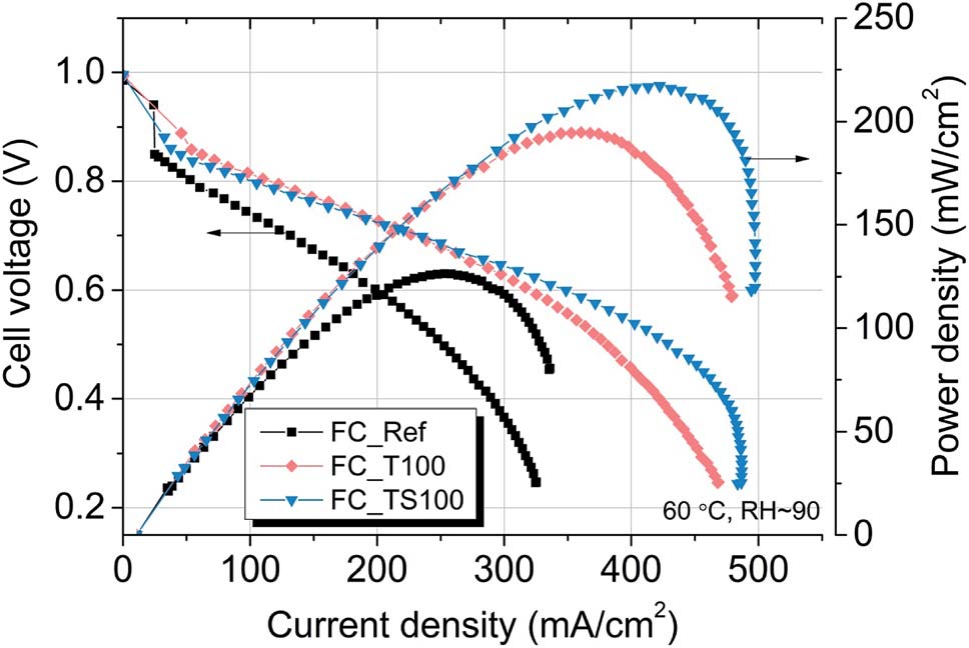
Polarization characteristics for TiO_2_–SiO_2_–VTMS and TiO_2_-modified fuel cells in comparison to Pt/carbon black/Nafion reference fuel cell (FC_Ref).

Improved rate of anodic reaction has a positive influence on the entire fuel cell performance assuming the kinetics at cathode side is elevated by much higher utilization of the catalyst. In the given experiment around 6-times higher platinum amount was used at cathode site (2.0 mg cm^−2^) than at anode (Table 1) in order to see the impact of modifiers on fuel cell operation. However, according to ref. 3 and 4 cathodic losses are still dominant due to extremely fast rate of HOR reaction in comparison to ORR. Nevertheless, performing direct voltage–current characteristics, the effect of anode modification was investigated with regard to reference sample which contains platinum-supported carbon electrode with Pt surface density of 0.54 mg cm^−2^. In Fig. 6 polarization curves are depicted as the electrical potential and power density functions of current density for reference fuel cell (FC_Ref) and fuel cells with TiO_2_–SiO_2_–VTMS- and TiO_2_-based anodes (FC_TS100 and FC_T100, respectively).

**Fig. 7:**
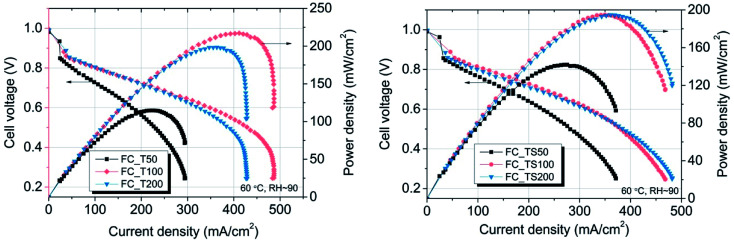
The differences in performance for given fuel cells having various concentrations of TiO_2_–SiO_2_–VTMS and TiO_2_ applied as anode modifiers.

Table 4 contains values of parameters that were calculated based on three different methods of *in situ* evaluation for fuel cells *i.e.* polarization curves, electrochemical impedance spectroscopy, cyclic voltammetry. Samples with TiO_2_–SiO_2_–VTMS- and TiO_2_ were additionally analyzed with regard to different concentrations of modifiers *i.e.* 50% and 200% of the amount applied for FC_TS100 and FC_T100 (marked as FC_T50 and FC_T200 for TiO_2_, and FC_TS50 and FC_TS200 for TiO_2_–SiO_2_–VTMS). The polarization curves that compare the influence of various modifiers concentrations are shown is Fig. 7. Each *V*–*I* characteristic was carried out maintaining the following configuration: hydrogen/air flow rate: 60/80 ml min^−1^, fuel cell fixture temperature 60 °C, cathode output relative humidity ∼90%, no dead-end nor back pressure. Both cathode and anode were humidified.

**Table 4:** Fuel cell parameters obtained in a series of electrochemical experiments: polarization curves (60 °C, Rh ∼ 90%), electrochemical impedance spectroscopy, cyclic voltammetry (25 °C)

Parameter/fuel cell	FC_Ref	FC_T100	FC_T50	FC_T200	FC_TS100	FC_TS50	FC_TS200
*J*_max_ [mA cm^−2^] (0.25 V)	325	**483**	295	429	469	372	482
*P*_max_ [mW cm^−2^]	126	**217**	115	198	195	142	195
Eff. HHV at *P*_max_ [%]	35.2	35.6	34.1	37.0	37.6	34.7	36.4
OCV^a^ [V]	0.99	0.99	0.98	0.99	0.99	0.99	1.00
*R*_electrode_ b [Ω cm^2^]	3.70	2.92	4.13	3.20	3.16	3.99	3.08
*R*_electrolyte_ c [Ω cm^2^]	0.36	0.26	0.35	0.23	0.25	0.26	0.25
ECSA^d^ [m^2^ g^−1^]	24.2	63.3	14.4	31.7	46.5	25.2	25.2

a OCV – open circuit voltage.

b *R*_electrode_ – total transfer resistance of mass and charge through the electrode.

c *R*_electrolyte_ – ionic resistance of the electrolyte.

d ECSA – electrochemical surface area.

**Fig. 8:**
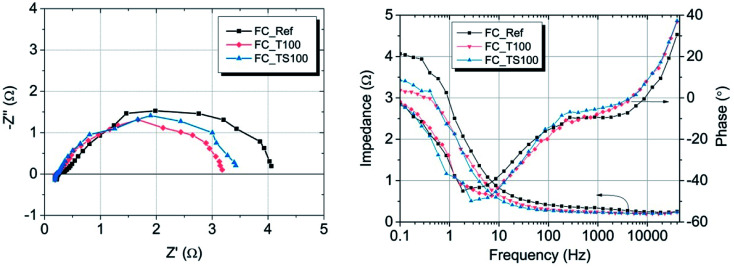
Nyquist (left) and Bode (right) plots of PEMFCs operated at 25 °C and Rh ∼ 90%, delivering hydrogen/air with flow rate 60/80 sccm, measurements without additional electrical load.

The results of experiment suggest that the amount of catalyst 0.34–0.54 mg cm^−2^ at anode and 2.0 mg cm^−2^ at cathode is sufficient at given conditions to investigate the anode behaviour of entire fuel cell despite sluggish kinetics of oxygen reduction reaction. The improvement of properties can be observed based on polarization curves carried out for particular fuel cells. Reference fuel cell (FC_Ref) is characterized by maximum power density of 126 mW cm^−2^ and current density (at 0.25 V) of 325 mA cm^−2^. Modification of anode catalyst support by addition of 12.4 wt% titanium dioxide (FC_T100) resulted in much higher power and current density (217 mW cm^−2^ and 483 mA cm^−2^, respectively). In contrast, after the addition of two times lower amount of TiO_2_ (6.6 wt%, FC_T50) to the anode the changes of reaction kinetics were not observed (115 mW cm^−2^, 295 mA cm^−2^), whereas double increase of modifier concentration (FC_T200) gave significant gain of performance (198 mW cm^−2^, 429 mA cm^−2^). FC_T200 behaviour is slightly worse than FC_T100 which shows that higher concentration of TiO_2_–SiO_2_–VTMS may not be advantageous perhaps due to poor electrical properties of SiO_2_. The efficiency at maximum power, calculated taking the higher heating value of hydrogen, shows that there are no significant differences of voltage losses between samples. The obtained values vary from 34.1 to 37.6%. Therefore, the improvement of fuel cells operation cannot be explained by the phenomena of activation losses nor ohmic losses decrease (due to ionic resistance of the membrane). There are significant differences in polarization concentrations, however these losses are mainly affecting the values of potential at high current density region which is beyond the nominal operating voltage range for fuel cells. The comparison of particular values of voltage losses were collected in Table 5. Activation polarization losses were calculated from the given equation 
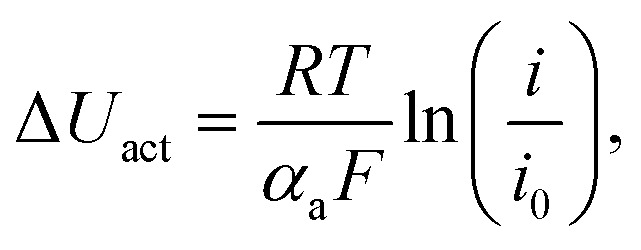
 where: *R* – gas constant, *T* – temperature, *α*_a_ – transfer coefficient, *F* – Faraday's constant, *i* – current density, *i*_0_ – exchange current density. Both parameters *α*_a_, *i*_0_ were taken based on the plot of Tafel equation. Ohmic losses are based on EIS experiment, where the values of resistance were obtained at high frequency region of Nyquist plot (Fig. 8).

**Table 5:** The values of voltage losses calculated on the basis of polarization curves and electrochemical impedance spectroscopy

Voltage losses	FC_Ref	FC_T100	FC_T50	FC_T200	FC_TS100	FC_TS50	FC_TS200
Activation [mV] at 0.2 A cm^−2^	462	413	480	421	413	460	426
Ohmic [mV] at 0.2 A cm^−2^	72	52	70	46	50	52	50
Concentration [mV] at *J*_max_	118	165	145	201	116	125	123

Concentration losses were found according to the following equation: 
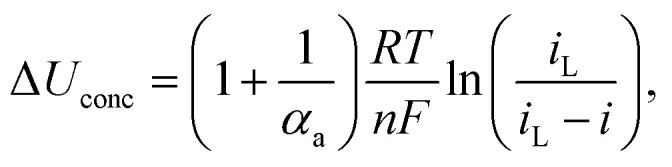
 where *n* – number of electrons per H_2_ molecule, *i*_L_ – limiting current density, calculated from the fitting of polarization curve using the function: 
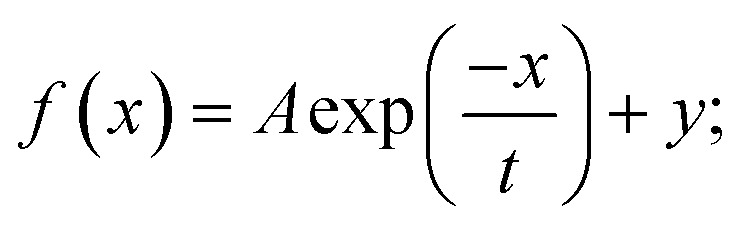
 (*A*, *t* and *y* coefficients were estimated using the method of the least squares).

According to the data in Table 5 activation losses were diminished for all modified fuel cells (around 413–426 mV) except FC_T50 and FC_TS50 in comparison to FC_Ref (462 mV). It is a direct evidence of modifiers influence. Ohmic losses calculated as electrolyte resistance contribution (EIS high frequency region) is low and comparable amongst samples. There is observed a significant gain of concentration losses for TiO_2_-modified fuel cells (145–201 mV) in contrast to other fuel cells (118–125 mV). It may be explained that at high current densities titanium dioxide interacts with mass and charge transfer phenomena and that interaction is triggered above certain value of current density. The relevant scenario is not observed in case of TiO_2_–SiO_2_–VTMS. The modification of catalyst support does not affect the open circuit voltage values. In case of all fuel cells the values of OCV are around 1.0 V.

#### 3.2.2. Mass and charge transfer resistance

To investigate the mass and charge transfer resistance for particular samples the electrochemical impedance spectroscopy (EIS) was applied. Fuel cells were supplied with hydrogen and air (60/80 sccm) while the cells were at a temperature of 25 °C and relative humidity of about 90%. The electrical load was not applied. All characteristics were measured in the frequency range from 40 kHz to 0.1 Hz with signal amplitude of 250 mV. Nyquist and Bode plots of EIS experiment for investigated fuel cells are shown in Fig. 8.

The resistance at high frequency region of Nyquist plot is a material property of the electrolyte (the sum of ionic resistance of membrane and Nafion electrolyte layers). All samples were manufactured according to the same method thus the values of the resistances are comparable (Table 4) and vary from 0.23 to 0.36 Ω cm^2^. Small fluctuation is mainly due to variations of electrolyte concentration at the anode side having fixed value of 33 wt% Nafion/Pt-carbon black regardless of the amount of modifier used. The resistance of mass and charge transfer which corresponds to the width of semicircle in Nyquist plot varies depending on fuel cells. According to Table 4 and at given conditions (open circuit voltage) the highest and comparable values were obtained for FC_Ref and fuel cells with low amount of modifying agent *i.e.* FC_TS50 and FC_T50 (3.70, 3.99 and 4.13 Ω cm^2^, respectively). This indicates that the low concentration of modifier doesn't contribute to the electrode transport phenomena or this contribution is negligible. However, noticeable influence is visible in case of the rest of the samples. The lowest values of resistance were obtained for FC_T100 (2.92 Ω cm^2^) while slightly higher in case of FC_T200 (3.20 Ω cm^2^). It is an evidence that titanium dioxide applied to the anode as a catalyst support facilitates the charge transport within the three phase boundary region or even increases the catalyst activity. TiO_2_ is a well-known semiconductor and photo-catalyst with good optical properties, capable of transferring protons across its crystal lattice.^29–31^ Relevant results were found in case of samples modified by TiO_2_–SiO_2_–VTMS, however the process that lead to the resistance mitigation is more complex. FC_TS100 and FC_TS200 with double concentration of modifying agent are characterized by similar transport resistance of 3.16 and 3.08 Ω cm^2^. In addition to the influence of TiO_2_, the observed improvement results also from the presence of silicon dioxide which provides the hydrophilic properties for the fuel cell electrode. The proper humidification of the electrode (especially anode) is of great importance in terms of increasing the power density. However, the usage of SiO_2_ is reasonable assuming its low concentration due to poor electrical conductivity. Vinyltrimethoxysilane acts as a coupling agent in TiO_2_–SiO_2_–VTMS. The compound is bifunctional, featuring both a vinyl group and hydrolytically sensitive ethoxysilyl groups. As such it also contributes to the hydrogen oxidation reaction rate *via* incorporation of additional water molecules (increased water uptake) and hydrolysis of TiO_2_ and SiO_2_ that influences the proton conduction mechanism within the electrolyte.

#### 3.2.3. Active surface area of catalyst mixture

**Fig. 9:**
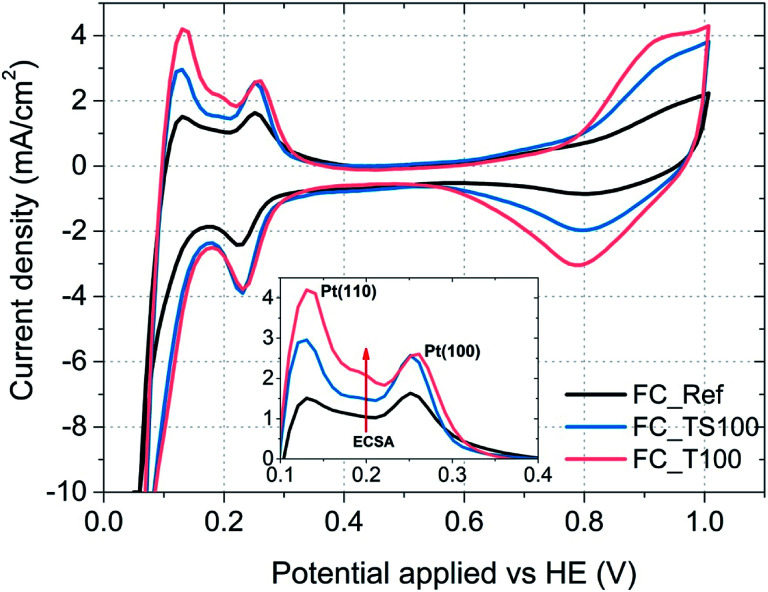
Cyclic voltammograms of fuel cells and characteristic desorption peaks assigned to the Pt(110) and Pt(100) crystal surface of the catalyst (inset); potential applied *versus* fuel cell anode.

**Fig. 10:**
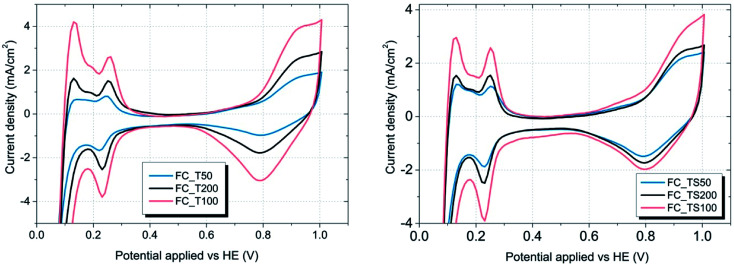
CV graphs of TiO_2_ and TiO_2_–SiO_2_–VTMS modified fuel cells – the influence of various concentrations.

Surface area of platinum catalyst that takes part in a hydrogen oxidation reaction is limited due to the electrode composition, various impurities or even the excess of water present within a catalyst region. The performance of fuel cells depends on this physical magnitude which is reflected by a parameter named as electrochemical surface area (ESCA). The higher the value of ECSA the better due to bigger utilization of a catalyst. To obtain the electrochemical surface area, cyclic voltammetry (CV) was used with the aid of a potentiostat. In this method particular fuel cells were scanned with the rate of 10 mV s^−1^ from 0 to 1 V, repeatedly and electric current response was recorded and plotted in the form of voltammograms (Fig. 9 and 10). One electrode (HE) was fed with hydrogen (50 ml min^−1^) working as counter and reference electrode while the other was supplied with nitrogen (50 ml min^−1^) acting as working electrode.

Electrochemical surface area of the catalyst was calculated based on the following equation: 
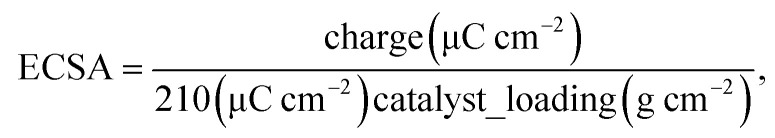
 where the amount of charge was obtained integrating the area underneath desorption peaks in the range from 100 to 400 mV.

As it is visible in Table 4, ECSA calculated for most of the samples is comparable (approx. 25 m^2^ g^−1^) except FC_T100 and FC_TS100 fuel cells (63.3 and 46.5 m^2^ g^−1^, respectively). Slightly elevated value of ECSA was achieved for FC_TS200, *i.e.* 31.7 m^2^ g^−1^. In case of TiO_2_–SiO_2_–VTMS-modified fuel cells the increase of electrochemical surface area might be explained based on conclusions taken from physical surface area and porosity analysis. TiO_2_–SiO_2_–VTMS scaled up the area and increased microporous fraction within which platinum particles were deposited. Higher three-phase boundary region contributes to adsorption and desorption processes of hydrogen which is also observed analyzing the height of CV peaks (reflecting the catalyst activity). Additionally, water distribution within the electrolyte of anode layer was increased as a consequence of SiO_2_ addition.

In case of FC_T100 the improvement of electrochemical surface area is more significant and cannot be explained by the change of anode physical structure – surface area and porosity were just slightly increased. It seems that TiO_2_ may participate in a hydrogen oxidation reaction *via* the contribution to the proton conduction mechanism and the increase of catalyst activity in terms of improvement of reaction rate. However, such a statement cannot be proven based on experiments conducted in this paper.

#### 3.2.4. Profitability estimation of new anode *vs.* Pt anode for PEMFCs

Despite recent successive development of various platinum-group metals (PGM)-free catalysts, Pt-based materials are still the most popular and commonly-used catalysts for fuel cell applications. There is observed continuous drop of required amount of platinum used in electrode layers. For instance, U.S. Department of Energy (DoE) set the goal of 0.125 g kW^−1^ of PGM catalyst by the year 2020 for low duty vehicles. It corresponds just to 11.25 g of platinum per midsize fuel cell electric vehicle (90 kW_gross_).^32^ The given value is comparable with the amount of PGM materials used for internal combustion engine vehicles however further reduction is needed for successful competition. Catalyst amount drop down to 0.088 g kW^−1^ is assumed by 2025 according to DoE. There are various methods to achieve such a target. The approach presented in this paper could be one of them. At given conditions our method allowed to decrease the amount of catalyst by circa 30%. For the 2020 DoE target it is relevant to reduction of Pt by 37.5 mg per 1 kW or 3.4 g per 90 kW vehicle. Replacement of carbon-based support materials alongside the improvement of electrode ionomer materials seem to be necessary for development of competitive fuel cell electric vehicles.

## Conclusions

Two chemical compounds were used for modification of anode support material for PEM fuel cells, *i.e.* TiO_2_ in an anatase phase and the hybrid material (TiO_2_–SiO_2_–VTMS) composed of titanium dioxide and silicon dioxide with vinyltrimethoxysilane acting as a crosslinking agent. TiO_2_–SiO_2_–VTMS is a good candidate for the modification of support material first of all due to its outstanding physical properties of high surface area and microporosity, and also because of its improved chemical durability and hydrophilicity, capable to stabilize catalyst nanoparticles and increase the rate of hydrogen oxidation reaction. TiO_2_–SiO_2_–VTMS applied in the amount of just 12.5 wt% leads to a threefold increase of catalyst layer surface area and it decreases the average pore size by 10 nm. In the consequence of modification, the electrochemical properties of fuel cell are improved, including enhancement of its electrochemical surface area, charge and mass transfer conductivity and the power density.

It was confirmed that pristine titanium dioxide can also be successfully utilized for fuel cell electrodes acting as a catalyst support. According to the results of this work, TiO_2_ added to anode of PEMFC has an influence on the electrochemical reaction that results in the improvement of the overall fuel cell performance. However, its poor surface area and limited porosity justify the usage of SiO_2_ in order to mitigate the typical catalyst degradation problems related for instance to platinum dissolution, migration and agglomeration phenomena. TiO_2_–SiO_2_–VTMS could be usable for PGM-free catalysts to solve their various problems.

## Conflicts of interest

There are no conflicts to declare.

## Supplementary Material
